# Long non-coding RNA DSCAM-AS1 contributes to the tumorigenesis of cervical cancer by targeting miR-877-5p/ATXN7L3 axis

**DOI:** 10.1042/BSR20192061

**Published:** 2020-01-03

**Authors:** Jie Liang, Shujuan Zhang, Wei Wang, Yan Xu, Atikan Kawuli, Jiaqi Lu, Xuemei Xiu

**Affiliations:** 1 Department of Gynaecology, Second People’s Hospital in Kashgar, Xinjiang Uyhur Autonomous Region, No.1, Health Road, Kashgar 844000, Xinjiang, China; 2 Department of Medical Oncology, Second People’s Hospital in Kashgar, Xinjiang Uyhur Autonomous Region, No.1, Health Road, Kashgar 844000, Xinjiang, China; 3 Department of Medical Oncology, The First People’s Hospital in Kashgar, Xinjiang Uyhur Autonomous Region, No.120, Yingbin Road, Kashgar 844000, Xinjiang, China

**Keywords:** ATXN7L3, cervical cancer, DSCAM-AS1, miR-877-5p

## Abstract

Cervical cancer (CC) is ranked as the fourth most common cancer that occurs in women universally, which normally causes pain in the lower belly. Plenty of studies have stated that the expression of long non-coding RNAs (lncRNAs) is linked to the cellular development of many kinds of cancers. DSCAM-AS1 has been reported to act as an oncogene in other cancer types and the aim of our study was to uncover the function and regulatory mechanism of DSCAM-AS1 in CC. In this research, our findings presented that DSCAM-AS1 expression was up-regulated in CC cells. DSCAM-AS1 led to the development of CC by enhancing cell proliferation, migration and invasion ability. DSCAM-AS1 was verified to combine with miR-877-5p and down-regulate the expression of miR-877-5p. Results also showed that ATXN7L3 was a downstream target gene of miR-877-5p and it was unfavorably modulated by miR-877-5p. Enhanced expression of ATXN7L3 counterbalanced the DSCAM-AS1 knockdown effect on the progression of CC. This was the first time to analyze the underlying regulatory mechanism of the oncogenic DSCAM-AS1. Our findings clarified that DSCAM-AS1 played as an oncogenic lncRNA by targeting miR-877-5p/ATXN7L3 axis to promote CC progression, which may provide insights into the prevention of CC.

## Introduction

Cervical cancer (CC) is regarded as one of the most successfully curable cancers, characterized by the abnormal growth of cervical cells [1]. Fundamentally, CC originates from infection with the virus called human papillomavirus (HPV) [2]. Women who have early sexual behavior, frequent sexual activity or birth control pills are more feasible to be infected by HPV, which means a high risk of getting CC [3]. The survival rate of CC patients at terminal stage is quite low as well. It is of great significance to delve into the regulatory mechanism in CC, so as to come up with new tactics to treat this disease later on.

LncRNAs, a type of non-coding RNAs with over 200 nucleotides, have attracted many scholars’ attention in regard to its diverse functions in cancers. For instance, CRNDE strengthens neuropathic pain through regulating the expression of miR-136 and IL6R in CCI rat models [4]. The LXR-623-induced long non-coding RNA (lncRNA) LINC01125 represses cell proliferation in breast cancer via the PTEN/AKT/p53 axis [5]. LncRNA ENST00000547547 suppresses the proliferation, invasion and migration of colorectal cancer cells [6]. LncRNA DSCAM-AS1 has been validated to be oncogenic in different sorts of cancers. For example, lncRNA DSCAM-AS1 is related to unsatisfactory clinical prognosis and contributes to melanoma development by sponging miR-136 [7]. LncRNA DSCAM-AS1 promotes non-small cell lung cancer by targeting BCL11A [8]. In recent years, an abundance of progress has been achieved in the identification of DSCAM-AS1 in cancer progression. Nonetheless, the function of DSCAM-AS1 in CC is still completely uncertain to us.

MiR-877-5p was proved to affect the progression of some cancers in several studies. For example, miR-877-5p inhibits cell proliferation, migration and invasion via targeting cyclin-dependent kinase 14 and acts as a biomarker for prognosis in hepatocellular carcinoma [9]. MiR-877-5p is involved in trovafloxacin-induced liver injury [10]. Yet, the exact function of miR-877-5p in CC has never been inspected heretofore.

During the present study, our objective was to analyze the underlying function of DSCAM-AS1 in CC progression. In the end, we substantiated that DSCAM-AS1 advanced the tumorigenesis of CC by functioning as a competing endogenous RNA (ceRNA) to up-regulate ATXN7L3 expression through miR-877-5p. These incredible findings cast light on the road for future cures in CC.

## Materials and methods

### Tissue samples

The study was carried out according to the Declaration of Helsinki and has been approved by the Ethical Committee of Second People’s Hospital in Kashgar, Xinjiang Uyhur Autonomous Region. No patients had experienced any form of tumor-specific treatment before diagnosis. Informed consent was obtained from all patients. Forty CC tissues and corresponding normal cervical tissues were provided by patients who underwent primary surgery in Second People’s Hospital in Kashgar, Xinjiang Uyhur Autonomous Region. All tissue samples were immediately frozen in liquid nitrogen and kept under the condition of −80°C.

### Cell lines and plasmids transfection

Human immortalized cervical epithelial cell line (H8) and CC cell lines (SiHa, HeLa, C-33A and CaSki) were bought from Cell Bank of Type Culture Collection (CBTCC, Shanghai, China) and were grown in DMEM culture medium (Gibco, Gaithersburg, MD, U.S.A.). All cell lines were cultivated with 10% fetal bovine serum (FBS) in 5% CO_2_ at the temperature of 37°C. To investigate the knockdown impact of DSCAM-AS1, sh-DSCAM-AS1#1/2 and sh-NC were acquired from Obio Technology (Shanghai, China). MiR-877-5p mimics and NC mimics were purchased from Tingzhou Biological Engineering Co., Ltd (Shanghai, China). Full ATXN7L3 was cloned to pcDNA3.1 plasmid to overexpress ATXN7L3. And primers for plasmid construction were provided in Supplementary Table S1. Above plasmids from ATTC (Manassas, VA, U.S.A.) were transfected into SiHa and CaSki cells with Lipofectamine 3000 Reagent (Thermo Fisher Scientific, CA, U.S.A.). All stably transfected cells were gathered for further study after 48 h.

### Quantitative real-time polymerase chain reaction

The extracted total cellular RNA from SiHa and CaSki cells by the TRIzol reagent (Invitrogen, Carlsbad, CA, U.S.A.) was quantitated with NanoDrop 2000 (Wilmington, DE, U.S.A.). Taqman Advanced microRNA (miRNA) cDNA Synthesis Kit was used to generate complementary DNA (cDNA) templates in strict accordance with the recommendations provided by supplier. SYBR Green Master Mix Kit and Light Cycler 480 II system (Roche, Shanghai, China) were applied for real-time PCR analysis. GAPDH or U6 as a loading control was used for RNA qualification. The application of 2^−ΔΔ*C*_t_^ comparative method was to analyze the relative RNA expression levels. Primers for quantitative real-time polymerase chain reaction (qRT-PCR) analysis were provided in Supplementary Table S1.

### Cell counting kit-8 assay

The transfected SiHa and CaSki cells were seeded (2 × 10^3^ cells/well) on the 96-well plates at the temperature of 37°C with 5% CO_2_. Following 0, 1, 2, 3 and 4 days of culture, 20 μl of cell counting kit-8 (CCK-8) solution (5 mg/ml) was put into the plates to assess modification in cell proliferation. Two hours later, absorbance at 450 nm was detected by a spectrophotometer.

### EdU assay

In line with instructions, the Cell-Light EdU imaging detection kit obtained from RiboBio (Guangzhou, China) was utilized for 5-ethynyl-2′-deoxyuridine (EdU) assay. The harvested SiHa and CaSki cells were rinsed twice in PBS and planted on to 96-well plates with 5000 cells in each well for 6 h. Then, EdU labeling medium was added and cultured for 1 h until cells adhered to the culture plates. Cells fixed in 4% formaldehyde were subjected to 0.5% Triton X-100 and Apollo reaction cocktail. Cells in each well were stained by DAPI and finally visualized under a fluorescence microscope.

### Wound healing assay

Forty-eight hours after transfection, the wounds were scratched on SiHa and CaSki cells by use of the ruler and pipette tip. Later medium was removed. After rinsing twice in PBS, cells were introduced into the medium with 1% FBS at 37°C with 5% CO_2_. Images of wound healing were taken with a microscope with the magnification of 50× at 0 and 24 h.

### Transwell invasion and migration assay

Cell invasive or migration ability was tested by the Transwell plates (24-well plate layout). Corning Costar Transwell plates with 8-μm pores were pre-covered with (for invasion) or without (for migration) Matrigel (BD Biosciences, San Jose, CA, U.S.A.) on the upper surface of the filter, and used for culturing SiHa or CaSki cells in non-serum medium at 37°C for as long as 3 h. The medium including 20% FBS was placed on to the lower chamber. After incubation for 48 h, the bottom invaded and migrated cells were fixed with 1% formaldehyde and marked with 0.1% Crystal Violet. Colored cells were monitored by a microscope (Nikon, Tokyo, Japan).

### RNA immunoprecipitation

In this assay, the EZMagna RIP Kit (Millipore, Billerica, MA, U.S.A.) was exercised. SiHa or CaSki cell lines in complete RNA immunoprecipitation (RIP) lysis buffer were cultivated with magnetic beads conjugated with Ago2 or control IgG at 4°C for 6 h. Once the beads were discarded, qRT-PCR analysis was performed for the purified RNA.

### RNA pull-down

SiHa or CaSki cell lines were transfected with biotin-labeled DSCAM-AS1, DSCAM-AS1-Mut or NC control, followed by incubation with streptavidin-plated magnetic beads (Ambion, Life Technologies). The pulled-down biotin-coupled complex was analyzed by qRT-PCR to determine the enrichment of miR-877-5p in bound fractions.

### Luciferase reporter assay

The DSCAM-AS1 or ATXN7L3 cDNA containing the predictive miR-877-5p binding sites was inserted into the pmirGLO plasmid (Promega, Madison, WI, U.S.A.) to form reporter DSCAM-AS1-Wt or ATXN7L3-Wt. DSCAM-AS1-Mut and ATXN7L3-Mut were generated using point mutations. SiHa or CaSki cells were co-transfected with the above wild-type or mutant type and miR-877-5p mimics or NC mimics. The Dual-Luciferase Reporter Assay System (Promega) was applied at 48 h after transfection as per the protocol.

### Western blotting

Total proteins of SiHa or CaSki cells were isolated in RIPA lysate buffer (Solarbio, Beijing, China), the concentration of which was measured with BCA kit (Beyotime, Shanghai, China). After mixing with loading buffer, samples were separated on 10% SDS/PAGE gels and transferred on to the PVDF membranes. To block the non-specific binding, membranes were cultured in 5% skimmed milk and treated with anti-ATXN7L3 (ab99947, 1/2000; Abcam, Cambridge, U.S.A.), anti-E-cadherin (ab15148; 1/1000; Abcam), anti-N-cadherin (ab76057; 1/1000; Abcam) and anti-GAPDH (ab37168; 1/1000; Abcam) antibodies at 4°C overnight, followed by treatment with IgG secondary antibody (Abcam). The bands were detected in ChemiDoc™ XRS+ System (Bio-Rad, U.S.A.) using enhanced chemiluminescence.

### Statistical analyses

All experimental data were exhibited as the mean ± standard deviation with no less than three independent samples. Both SPSS 22.0 statistical software package and GraphPad Prism 7.0 were utilized in the present study for statistical analyses. Student’s *t* test or one-way ANOVA was conducted for different analyses in two or more groups, with the significance of *P*<0.05.

## Results

### DSCAM-AS1 expression is remarkably up-regulated in cervix cancer

**Figure 1:**
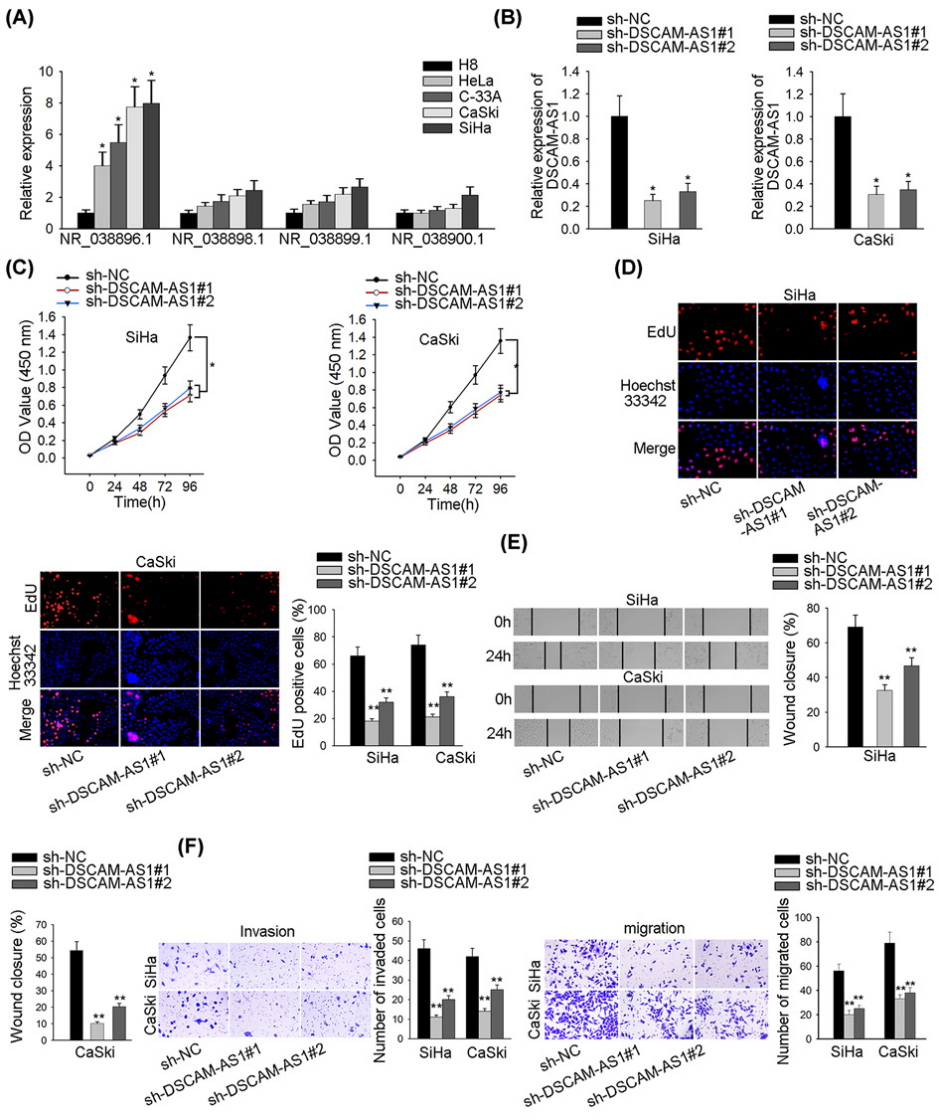
DSCAM-AS1 is immensely up-regulated and promotes cellular proliferation and metastasis in CC (**A**) DSCAM-AS1 expression levels in CC cells and related normal cells. (**B**) DSCAM-AS1 knockdown efficiency was tested by qRT-PCR after cells were transfected with sh-DSCAM-AS1#1 and sh-DSCAM-AS1#2. (**C,D**) CCK-8 and EdU proliferation analyses with DSCAM-AS1 knocked down in SiHa and CaSki cells. (**E,F**) Wound healing, transwell invasion and migration experiments were performed with DSCAM-AS1 deduction. *P<0.05, **P<0.01.

Even though DSCAM-AS1 has been demonstrated to stimulate certain sorts of cancers [8,11,12], its role in CC is yet to be discovered. The qRT-PCR analysis showed that DSCAM-AS1 was most highly expressed in transcript NR_038896.1 in CC cell lines (SiHa, HeLa, C-33A and CaSki), rather than in the corresponding normal cell line (H8) (Figure 1A). DSCAM-AS1 was silenced by transfection with sh-DSCAM-AS1#1/#2 and the effectiveness of the silencing was validated by qRT-PCR (Figure 1B). Proliferation assays CCK-8 and EdU depicted that DSCAM-AS1 expression could accelerate the cell proliferation in CC (Figure 1C,D). As in Figure 1E,F, DSCAM-AS1 down-regulation suppressed the migration and invasion in SiHa and CaSki cells; in addition, knockdown of DSCAM-AS1 enhanced the protein level of E-cadherin, but declined that of N-cadherin (Supplementary Figure S1A), indicating that DSCAM-AS1 promoted the cellular metastasis activity. In summary, DSCAM-AS1 had great expression levels in the CC cell lines, which improved a whole bunch of activities like proliferation, migration and invasion in CC.

### DSCAM-AS1 combines with miR-877-5p in cervix cancer

**Figure 2:**
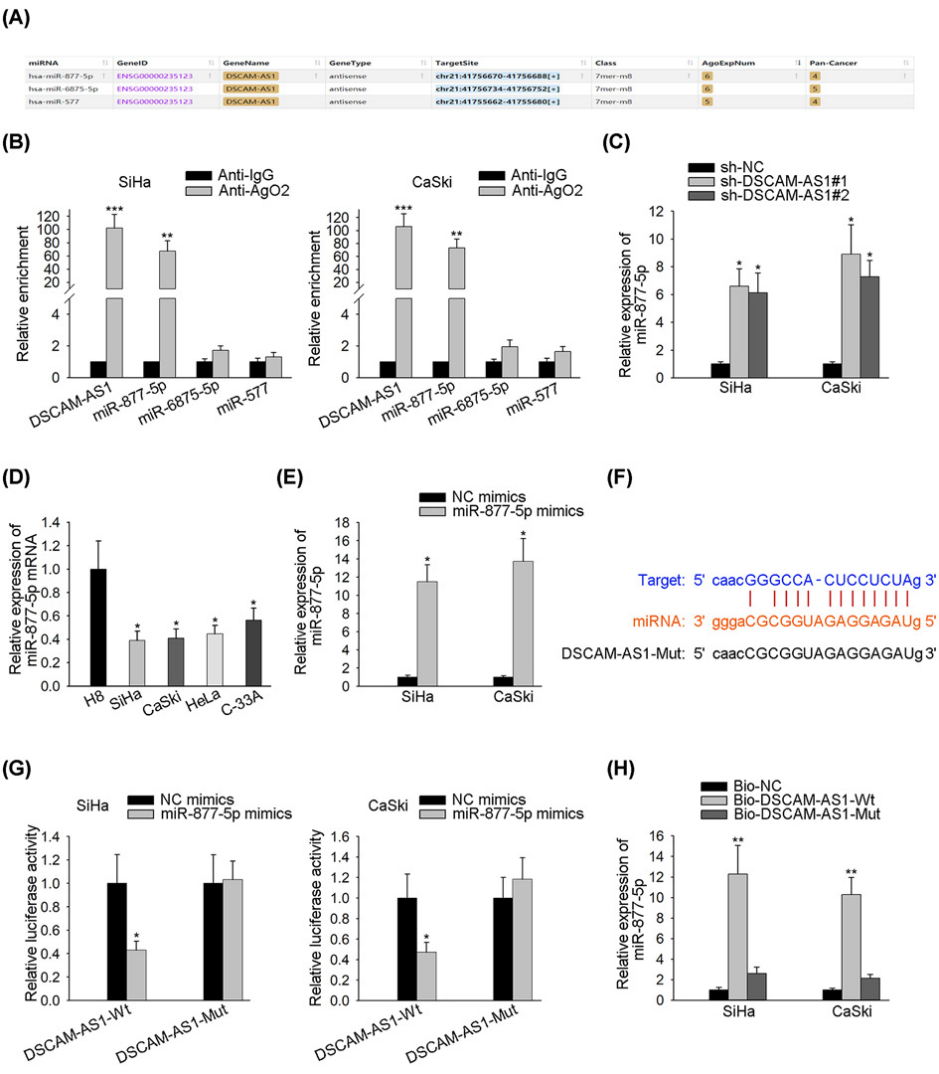
DSCAM-AS1 sponges miR-877-5p in CC cells (**A**) The starBase v2.0 database matched certain miRNAs that might bind with DSCAM-AS1. (**B**) Relative enrichment of the aforementioned miRNAs and DSCAM-AS1 in RISC were tested by RIP assay using Ago2 antibody and anti-IgG_._ (**C**) The impact of DSCAM-AS1 on miR-877-5p expression in SiHa and CaSki cells was detected by qRT-PCR. (**D**) qRT-PCR tested miR-877-5p expression levels in CC cells and relative normal cells. (**E**) The effectiveness of overexpressing miR-877-5p with miR-877-5p mimics was evaluated by qRT-PCR. (**F**) Bioinformatics predicted specific sites on miR-877-5p that bind to DSCAM-AS1. (**G**) Luciferase activities of DSCAM-AS1-Wt and DSCAM-AS1-Mut in a luciferase reporter assay after miR-877-5p overexpression. (**H**) During an RNA pull-down assay, miR-877-5p expression was perceived by the biotinylated DSCAM-AS1 pull down in CC cells. *P<0.05, **P<0.01.

Now that we had investigated the function of DSCAM-AS1 in CC, it was necessary to analyze the interaction between DSCAM-AS1 and miRNA. In light of the starBase v2.0 database, we obtained that there might be a few miRNAs (miR-877-5p, miR-6875-5p and miR-577) that had the likelihood to combine with DSCAM-AS1 (Figure 2A). RIP assay showed DSCAM-AS1 and miR-877-5p were enriched in the RNA-induced silencing complex (RISC) combination, thereby proving that DSCAM-AS1 was bound with miR-877-5p (Figure 2B). It was also determined that silence of DSCAM-AS1 would increase the expression levels of miR-877-5p (Figure 2C). Figure 2D implied that miR-877-5p was underexpressed in the CC cells, as compared with that in normal cells. Figure 2E displayed that miR-877-5p mimics were authenticated to have the effectiveness of overexpressing miR-877-5p. Additionally, bioinformatics hypothesized there were specific potential binding sites on DSCAM-AS1, with which miR-877-5p could bind (Figure 2F). Luciferase reporter and RNA pull-down assays validated the previous hypothesis that DSCAM-AS1 bound with miR-877-5p (Figure 2G,H). To recap, DSCAM-AS1 sponged miR-877-5p in CC, and the expression of miR-877-5p was negatively regulated by DSCAM-AS1.

### MiR-877-5p binds to ATXN7L3 in cervix cancer

**Figure 3:**
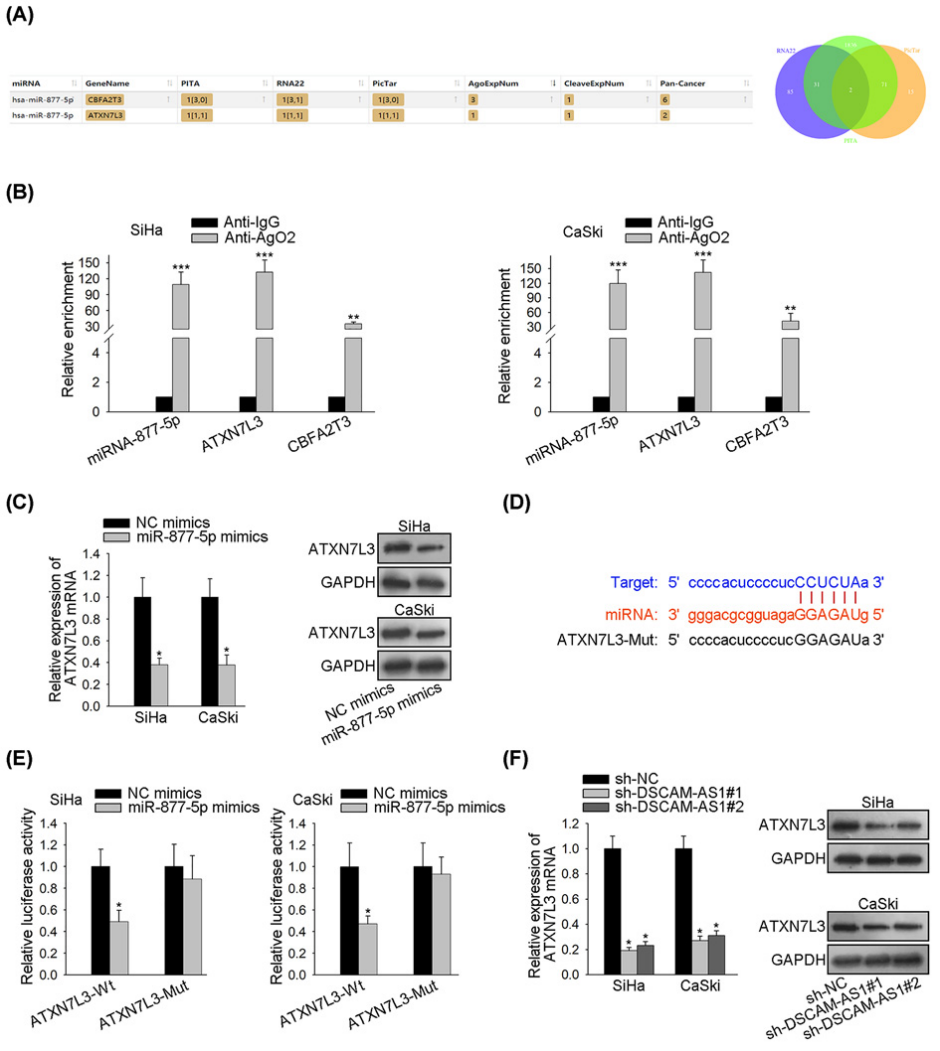
MiR-877-5p combines with ATXN7L3 in CC (**A**) Potential target mRNAs (CBFA2T3 and ATXN7L3) for miR-877-5p were discovered by databases PITA, RNA22 and PicTar. (**B**) RIP assays were carried out by adding antibodies of Ago2 and IgG, and relevant enrichment of miR-877-5p and mRNAs in SiHa and CaSki cells were examined by the qRT-PCR assay. (**C**) qRT-PCR observed the alteration in ATXN7L3 mRNA expression level after miRNA-877-5p was overexpressed. (**D**) Binding sites on ATXN7L3 mRNA for miR-877-5p were speculated with bioinformatics. (**E**) Luciferase activities of ATXN7L3-Wt and ATXN7L3-Mut were observed by luciferase reporter assay after enhancing the miR-877-5p expression. (**F**) qRT-PCR and Western blot assays were applied to figure out the relative expression of ATXN7L3 after DSCAM-AS1 deficiency. GAPDH was an internal control. *P<0.05, **P<0.01, ***P<0.001.

To discover the gene miR-877-5p targeted, database (PITA, RNA22 and PicTar) matched two potential target messenger RNAs (mRNAs) (CBFA2T3 and ATXN7L3) for miR-877-5p (Figure 3A). By analyzing the composition of the RISC, it was discovered that the enrichment of ATXN7L3 and miR-877-5p was strikingly higher than that of CBFA2T3 and miR-877-5p (Figure 3B). In Figure 3C, overexpressing miRNA-877-5p tremendously inhibited the expression of ATXN7L3 mRNA as well as its transcriptional protein. Bioinformatics was applied again to predict the binding sites miR-877-5p had on ATXN7L3 mRNA (Figure 3D). And, the following luciferase reporter assay confirmed this prediction that miR-877-5p bound to ATXN7L3 mRNA (Figure 3E). Besides, results from qRT-PCR and Western blotting suggested that DSCAM-AS1 shortage lowered the ATXN7L3 mRNA and protein expression levels (Figure 3F). To sum up, miR-877-5p bound to ATXN7L3 mRNA and miR-877-5p could negatively work on the expression of ATXN7L3.

### Enhanced expression of ATXN7L3 counterbalances the DSCAM-AS1 insufficiency effect on the development of CC

**Figure 4:**
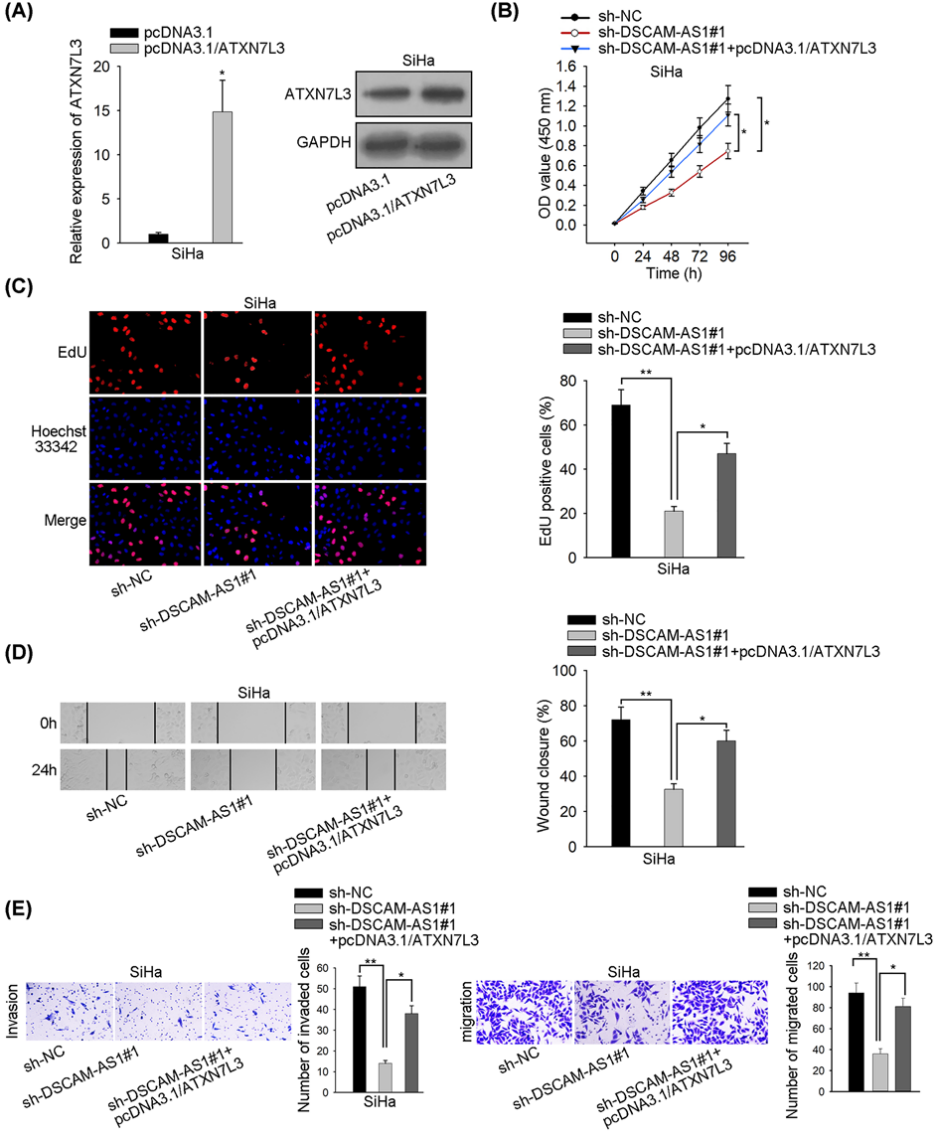
ATXN7L3 overexpression partly retrieved DSCAM-AS1 influence on CC progression (**A**) ATXN7L3 mRNA and protein overexpression effectiveness were assessed by qRT-PCR and Western blot assays. (**B,C**) The change in cell proliferation ability (after silencing DSCAM-AS1 and overexpressing ATXN7L3) was estimated by CCK-8 and EdU analyses. (**D,E**) Cellular metastasis capability subsequent to DSCAM-AS1 knockdown and ATXN7L3 overexpression was evaluated by wound healing, transwell invasion and migration experiments. GAPDH was utilized as an internal control. *P<0.05, **P<0.01.

On the foundation of earlier experiments, it needed further exploration about whether the oncogenic regulatory mechanism of DSCAM-AS1 was through the miR-877-5p/ATXN7L3 tunnel. As shown in Figure 4A, pcDNA3.1/ATXN7L3 was used to elevate the expression of ATXN7L3 and the overexpression effect functioned as expected. Initially, results unveiled that DSCAM-AS1#1 insufficiency prohibited cellular growth in CC. In rescue experiments, a rise in the number of live CC cells was beheld by CCK-8 and EdU assays after overexpressing ATXN7L3 (Figure 4B,C). The data obtained in wound healing, Transwell and Western blot assays implied that enhanced expression levels of ATXN7L3 also countervailed the DSCAM-AS1 knockdown effect on invasive or migratory capability in CC (Figure 4D,E). In addition, EMT-related proteins were assessed and the results reflected that the decreased level of N-cadherin and increased level of E-cadherin induced by silenced DSCAM-AS1 was rescued by the up-regulation of ATXN7L3 (Supplementary Figure S1B). Finally, the expression level of DSCAM-AS1, miR-877-5p and ATXN7L3 were separately detected in CC tissues and corresponding normal tissues. Unsurprisingly, we observed that both DSCAM-AS1 and ATXN7L3 were expressed higher in CC tissues, which were opposite with miR-877-5p (Supplementary Figure S2A–C). Ultimately, we could reach a conclusion that DSCAM-AS1 accelerated the progression of CC by targeting the miR-877-5p/ATXN7L3 axis.

## Discussion

CC is considered the second most commonly diagnosed cancer and the third prevailing cause of cancer deaths across female group, specifically with a high occurrence in developing countries [13,14]. So far, numerous lncRNAs have been believed to have the role of promoting the growth of CC. For example, lncRNA SOX21-AS1 accelerates CC development by sponging miR-7 and targeting VDAC1 [15]. LncRNA SBF2-AS1 contributes to the development of CC by modulating the miR-361-5p/FOXM1 axis [16]. LncRNA RP11-552M11.4 is conducive to oncogenesis and development of CC through regulating miR-3941/ATF1 signaling pathway [17]. Up-regulated expression of lncRNA CASC15 predicts bad prognosis and relates to tumor growth in CC [18]. In this case, whether DSCAM-AS1 has the same function and plays as an oncogene in CC remains mysterious. In the present study, we found out that DSCAM-AS1 expression was greatly up-regulated in CC cells and low expression level of DSCAM-AS1 prevented cell growth invasion and migration, suggesting that DSCAM-AS1 had an encouraging effect on the CC development.

Here, we recognized the regulatory mechanism behind the DSCAM-AS1 up-regulation in CC cells. MiRNAs are known as another kind of non-coding RNAs that have a length of approximately 22 nucleotides. It was registered by some studies that lncRNAs act as ceRNAs to hamper miRNAs from binding with downstream mRNAs. For instance, lncRNA CASC2 acts as a ceRNA of miR-21 to up-regulate the expression of PDCD4 in oral squamous cell carcinoma [19]. LncRNA LINC01234-induced by SP1 as a ceRNA promotes non-small cell lung cancer by modulating OTUB1 [20]. LncRNA GAS5 serves as a ceRNA for miR-21 to repress PDGF-bb-activated proliferation and migration of vascular smooth muscle cells [21]. LncRNA SBF2-AS1 acts as a ceRNA to affect acute myeloid leukemia cell proliferation through sponging with miR-188-5p [22]. LncRNA AGAP2-AS1, functioning as a ceRNA, up-regulates the expression of ANXA11 via binding with miR-16-5p and promotes cell proliferation and metastasis in hepatocellular carcinoma [23]. In our analysis, we discovered that DSCAM-AS1 bound with miR-877-5p and DSCAM-AS1 modulated the expression of miR-877-5p in a negative manner in CC.

To our knowledge, lncRNAs, miRNAs and mRNAs could comprise the so-called ceRNA network in many studies. For instance, lncRNAs, mRNAs and miRNAs compose a ceRNA network in laryngeal squamous cell carcinoma and are identified as potential biomarkers [24]. Previous study has indicated that ATXN7L3 exerts an important influence on cancer development. For example, ATXN7L3 and ENY2 stimulate activity of multiple H2B deubiquitinases that play a key role in cellular proliferation and tumor growth [25]. However, whether ATXN7L3 can promote tumor development in CC is undiscovered yet. Our results displayed that ATXN7L3 was the downstream target of miR-877-5p. MiR-877-5p could also bind to ATXN7L3 and negatively adjusted the expression levels of ATXN7L3. At last, we found out that overexpressing ATXN7L3 could counteract the restraining impact of DSCAM-AS1 knockdown on the proliferation, migration and invasion of CC.

In outline, our work corroborated that DSCAM-AS1 facilitates the tumor growth of CC by targeting miR-877-5p/ATXN7L3 axis, which could deliver unbelievable methods for CC treatment.

## Supplementary Material

Supplementary Figures S1-S2

Supplementary Table S1

## Abbreviations

ATXN7L3: ataxin 7 like 3
CC: cervical cancer
CCK-8: cell counting kit-8
cDNA: complementary DNA
ceRNA: competing endogenous RNA
DSCAM-AS1: DS cell adhesion molecule antisense RNA 1
EdU: 5-ethynyl-2′-deoxyuridine
FBS: fetal bovine serum
HPV: human papillomavirus
lncRNA: long non-coding RNA
miRNA: microRNA
mRNA: messenger RNA
qRT-PCR: quantitative real-time polymerase chain reaction
RIP: RNA immunoprecipitation
RISC: RNA-induced silencing complex
ShRNA: short hairpin RNA
