# Supplementary material for: Long non-coding RNA DSCAM-AS1 contributes to the tumorigenesis of cervical cancer by targeting miR-877-5p/ATXN7L3 axis
Source: Biosci Rep. 2020 Jan 3;40(1):BSR20192061. doi: 10.1042/BSR20192061 (PMC6944662; doi:10.1042/BSR20192061)
Supplement: Supplementary Figures S1-S2 [file BSR-2019-2061_supp.pdf]

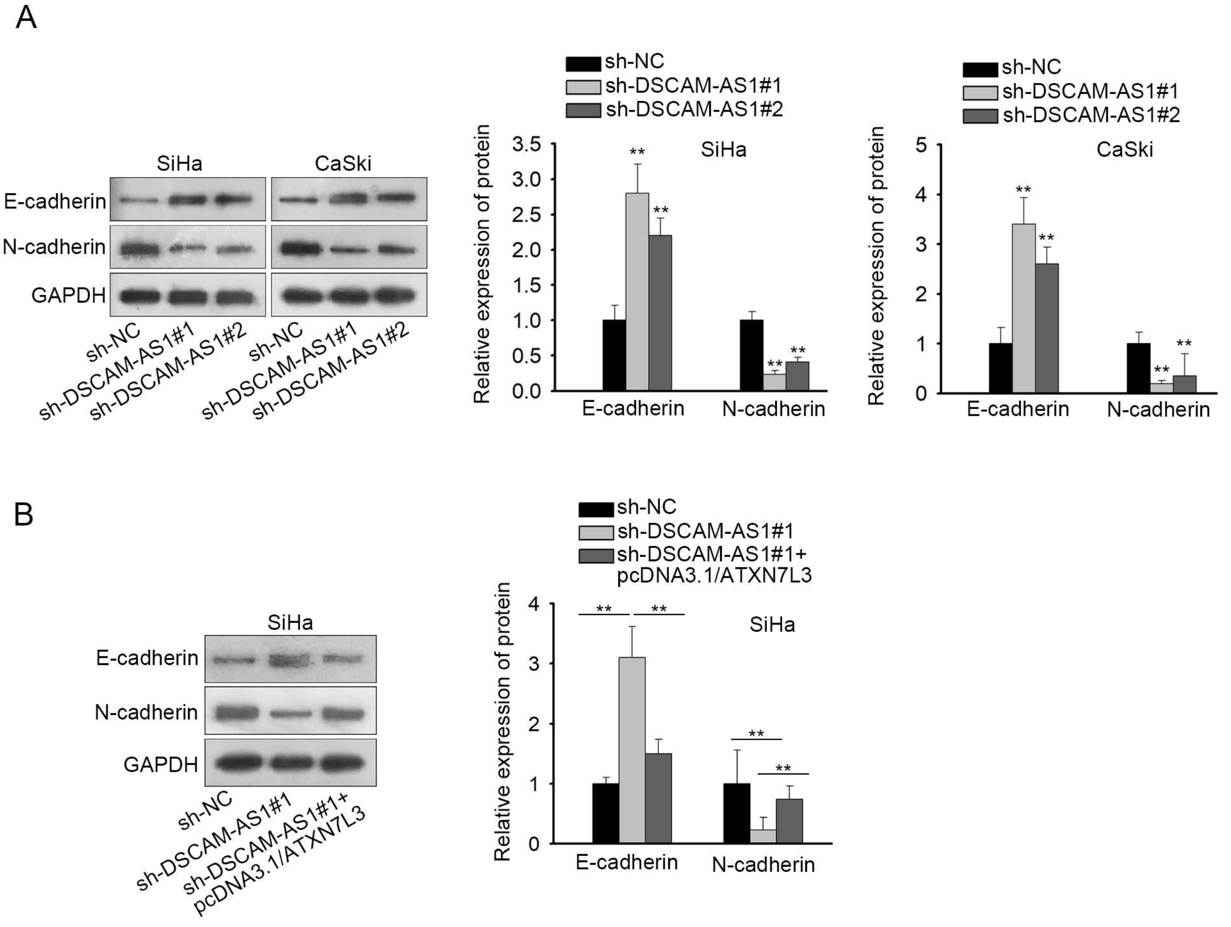
A
SiHa
CaSki
E-cadherin
N-cadherin
GAPDH
sh-NC
sh-DSCAM-AS1#1
sh-DSCAM-AS1#2
sh-NC
sh-DSCAM-AS1#1
sh-DSCAM-AS1#2
Relative expression of protein
SiHa
E-cadherin
N-cadherin
Relative expression of protein
CaSki
E-cadherin
N-cadherin
B
SiHa
E-cadherin
N-cadherin
GAPDH
sh-NC
sh-DSCAM-AS1#1
sh-DSCAM-AS1#1+pcDNA3.1/ATXN7L3
Relative expression of protein
SiHa
E-cadherin
N-cadherin

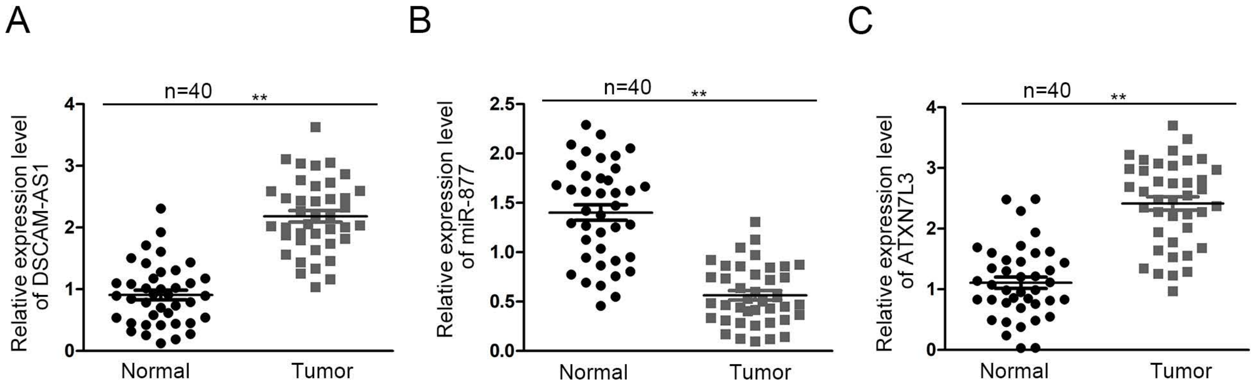
A
B
C
n=40
**
Relative expression level of DSCAM-AS1
4
3
2
1
0
Normal
Tumor
n=40
**
Relative expression level of miR-877
2.5
2.0
1.5
1.0
0.5
0.0
Normal
Tumor
n=40
**
Relative expression level of ATXN7L3
4
3
2
1
0
Normal
Tumor

Figure S1 (A-B) Western blot assays detected the expression level of E-cadherin and N-cadherin in differently transfected groups. ** $P< 0.01$.

Figure S2 (A-C) qRT-PCR measured the expression of DSCAM-AS1, miR-877-5p and ATXN7L3 in CC and normal tissues (n=40). ** $P< 0.01$.
